# Mixed Reality Platforms in Telehealth Delivery: Scoping Review

**DOI:** 10.2196/42709

**Published:** 2023-03-24

**Authors:** Hemendra Worlikar, Sean Coleman, Jack Kelly, Sadhbh O’Connor, Aoife Murray, Terri McVeigh, Jennifer Doran, Ian McCabe, Derek O'Keeffe

**Affiliations:** 1 Health Innovation Via Engineering Laboratory Cúram Science Foundation Ireland Research Centre for Medical Devices University of Galway Galway Ireland; 2 Department of Medicine, University Hospital Galway Galway Ireland; 3 Cancer Genetics Unit, The Royal Marsden National Health Service Foundation Trust London United Kingdom; 4 School of Medicine, College of Medicine Nursing and Health Sciences University of Galway Galway Ireland; 5 Lero, Science Foundation Ireland Centre for Software Research University of Limerick Limerick Ireland

**Keywords:** augmented reality, virtual reality, mixed realities, telemedicine, eHealth, mobile health, mHealth

## Abstract

**Background:**

The distinctive features of the digital reality platforms, namely augmented reality (AR), virtual reality (VR), and mixed reality (MR) have extended to medical education, training, simulation, and patient care. Furthermore, this digital reality technology seamlessly merges with information and communication technology creating an enriched telehealth ecosystem. This review provides a composite overview of the prospects of telehealth delivered using the MR platform in clinical settings.

**Objective:**

This review identifies various clinical applications of high-fidelity digital display technology, namely AR, VR, and MR, delivered using telehealth capabilities. Next, the review focuses on the technical characteristics, hardware, and software technologies used in the composition of AR, VR, and MR in telehealth.

**Methods:**

We conducted a scoping review using the methodological framework and reporting design using the PRISMA-ScR (Preferred Reporting Items for Systematic Reviews and Meta-Analyses Extension for Scoping Reviews) guidelines. Full-length articles in English were obtained from the Embase, PubMed, and Web of Science databases. The search protocol was based on the following keywords and Medical Subject Headings to obtain relevant results: “augmented reality,” “virtual reality,” “mixed-reality,” “telemedicine,” “telehealth,” and “digital health.” A predefined inclusion-exclusion criterion was developed in filtering the obtained results and the final selection of the articles, followed by data extraction and construction of the review.

**Results:**

We identified 4407 articles, of which 320 were eligible for full-text screening. A total of 134 full-text articles were included in the review. Telerehabilitation, telementoring, teleconsultation, telemonitoring, telepsychiatry, telesurgery, and telediagnosis were the segments of the telehealth division that explored the use of AR, VR, and MR platforms. Telerehabilitation using VR was the most commonly recurring segment in the included studies. AR and MR has been mainly used for telementoring and teleconsultation. The most important technical features of digital reality technology to emerge with telehealth were virtual environment, exergaming, 3D avatars, telepresence, anchoring annotations, and first-person viewpoint. Different arrangements of technology—3D modeling and viewing tools, communication and streaming platforms, file transfer and sharing platforms, sensors, high-fidelity displays, and controllers—formed the basis of most systems.

**Conclusions:**

This review constitutes a recent overview of the evolving digital AR and VR in various clinical applications using the telehealth setup. This combination of telehealth with AR, VR, and MR allows for remote facilitation of clinical expertise and further development of home-based treatment. This review explores the rapidly growing suite of technologies available to users within the digital health sector and examines the opportunities and challenges they present.

## Introduction

### Background

The term telemedicine refers to the provision of clinical health care services over a distance through information and communication technology (ICT) channels. Telemedicine overcomes geographical barriers in facilitating remote medical services. Building on this, the concept of telehealth extends to include continuing health education, research, and evaluation by medical professionals, all while promoting the health outcomes of individuals and communities [1]. Telehealth broadly encompasses the delivery of remote health-related services, including nonclinical services such as medical provider training; medical education; public health education; administrative meetings; and electronic exchange of clinical data enabling diagnosis, evaluation, consultation, treatment, and care management. The term telehealth has evolved as available technologies have improved, such that the term “digital health” is now often used as a more inclusive term reflecting the application of various different types of technologies and telecommunications systems in health care delivery. Digital health platforms can be either provider-to-provider or direct-to-consumer systems supported by the ICT infrastructure [2,3]. The telehealth sector has seen an effective increase in the past few years and has grown exponentially because of COVID-19 pandemic restrictions. According to the report published by Fortune Business Insights, the global telehealth market size was estimated at around US $144.38 billion in 2020 and is likely to reach US $636.38 billion by 2028 [4].

**Figure 1:**
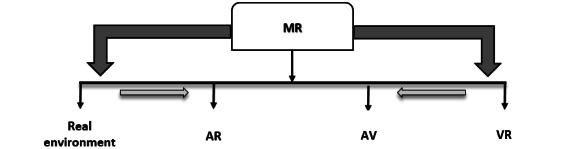
Representation of reality-virtuality continuum by Milgram et al [5]. AR: augmented reality; AV: augmented virtuality; MR: mixed reality; VR: virtual reality.

From the reality-virtuality continuum model, according to Milgram et al [5] (as seen in Figure 1), the real environment is that which is viewed without any overlay of the computer-generated entity, while at the opposite end of this continuum, immersive virtual reality (VR) is observed as completely enhanced computer-generated environments viewed through a head-mounted display unit. In the augmented reality (AR)–based display, digital information or entities are overlaid in the real environment, such that different aspects of reality are observed between the real and virtual environment. These augmentation-based realities can be discovered by optical see-through head-mounted displays (HMDs), mobile phones, tablets, or computer monitors [5].

In AR technology, the digitally created data directly coincide with the user’s real-world environment, where the user can see the computer-generated 2D or 3D entities such as holograms. The virtual entities superimposed or mapped onto the real-world space are typically rendered using optical see-through display such as HMDs or mobile-based devices, also allowing for stereoscopic visualization. The next most advanced form of reality platform, the mixed reality (MR), follows the footstep of AR and allows interaction with these virtual entities by using hand gesture inputs, gaze recognition, or controllers. The VR platform is a completely enhanced digital representation featuring a 2D or 3D virtual environment or objects that can replicate real-life surroundings. VR provides engaging sensory perceptions for both visual and acoustic stimulation. Immersive VR relies on headsets or stand-alone VR devices, whereas nonimmersive VR relies on the monitor display [6].

The introduction of VR and AR technologies in medicine has been focused on clinical-related research. The key areas incorporating this digital reality are surgery, psychology, neurological condition, rehabilitation, and medical educational [7]. The 3D picturing capabilities of the VR- and AR-based platforms have been sought for applications in the visualization of scientific experimental imaging data, tools for surgical planning and studying anatomy, and other collaborative interfaces for education and telehealth [8]. Surgical simulation has distinctively used digital reality, while VR is principally used for visual and haptic rendering, whereas AR and MR were predominantly positioned for the tracking system and graphical rendering, with the latter being used in a real surgical setting [9]. The usefulness of VR education and training using simulation methods for nursing students was comparable with the standard models of education and training on the outcomes of skills, confidence, satisfaction, and performance time [10]. The current prospects of AR software applications in medical criteria are treatment and training based [11]. Surgical development using an MR platform has been linked as a predominant utilization tool for training and simulation technology, advanced imaging and navigation, and broadening the extent of clinical application. Recently, MR has been adapted to neurosurgery, otolaryngology, ophthalmology, urology, and dentistry [12]. Digital reality technology has been incorporated into the preoperative surgical planning for several cranial-based applications for the neurosurgical subspecialty [13]. VR-based exposure therapy is used for various psychiatric disorders such as anxiety, trauma and stress, neurocognitive disorders, and several mental disorders. The effects of VR have been studied to have long-lasting positive outcomes for the treatment [14]. VR-based training has been effective in the improvement of executive limb function and cognitive function in patients with stroke [15,16].

### Objectives

Many published studies have reviewed the use of AR and VR capabilities in medical research and practice and have not detailed its implication in telehealth, thus addressing this research gap. This systematic scoping review provides an overview of the prospects of AR and VR applications delivered using telehealth platforms in clinical settings. This review offers end users and providers an update of the current use of AR, VR, and MR effectively in telehealth delivery and highlights the prospects of such technologies in the future. This review aims to explore the following research questions:

- What clinical specialties have incorporated digital reality platforms such as AR, VR, or MR exclusively with telehealth?
- What are the different hardware and software technology formats used in AR, VR, or MR within telehealth?
- Which important technical features of AR and VR have been used in telehealth?

## Methods

### Overview

This scoping review used the framework of the PRISMA-ScR (Preferred Reporting Items for Systematic Reviews and Meta-Analyses extension for Scoping Reviews) guidelines [17]. Included studies from the database were solely concerned with the application of high-fidelity simulation technology such as AR, VR, or MR exclusively delivered via the telehealth platform. The study has no written or published protocol.

### Database and Search Strategy

Articles from Embase, PubMed, and Web of Science were explored to obtain relative pieces of evidence. An exploded search strategy string was developed with the support of a university librarian. The search string included appropriate keywords and Medical Subject Headings terms—“augmented reality,” “virtual reality,” “mixed reality,” “extended reality,” “telemedicine,” “telehealth,” “m-Health,” “e-Health,” and “digital health.” The search strategy was initially developed on the Embase database and replicated across the other databases using predefined filtering techniques. The entire search strategy can be seen in Multimedia Appendix 1.

### Eligibility Criteria

The studies included must satisfy the active component use of AR, VR, or MR delivered via telehealth approaches and should have been published between the years 2016 and 2021, since such devices with this technology format became commercially available, marked in reference to the release date for the first-generation Microsoft HoloLens [18]. The collaboration aspect of AR and MR technology into social or digital communication avenues could be observed during the same period [19]. Telemedicine or telehealth includes a broad spectrum of health care delivery, including education prospects; however, this review will focus on clinical aspects, including simulation. Only full-length text articles available on the web in the English language were included. Full-length text from peer-reviewed articles such as randomized controlled trials, feasibility studies, exploratory studies, narrative reviews, systematic reviews, case and cohort studies, book sections, and technical reports was considered eligible for inclusion. Any studies highlighting the mentioned technology for gaming, entertainment, or medical education were excluded. Correspondence papers, letters, conference abstracts (no full texts), editorial, commentary, poster presentations, and gray literature were also excluded from this review.

### Study Selection and Data Extraction

The papers obtained from the applied search strategy from the information databases were imported to the reference manager EndNote 20 library, and duplicates were discarded [20]. Three researchers (HW, SC, and JK) performed initial screenings based on titles, abstracts, and keyword searches. Author HW conducted eligibility criteria and full-text screening. The selected studies were then reviewed based on the article type, study design, clinical condition addressed in the study, mode of telehealth communication, acceptance criteria, and the hardware and software used in the studies for the guidance for data synthesis. Finally, the relevant information from the studies was tabulated into an Excel (Microsoft Corp) spreadsheet, and a descriptive synthesis of the data was generated. In our review, we summarized and grouped the various telehealth branches using digital reality platforms for the various clinical condition based on descriptive statistical findings for the included studies. Different facets of the digital reality technology were detailed for its application in clinical research.

## Results

### Overview

**Figure 2:**
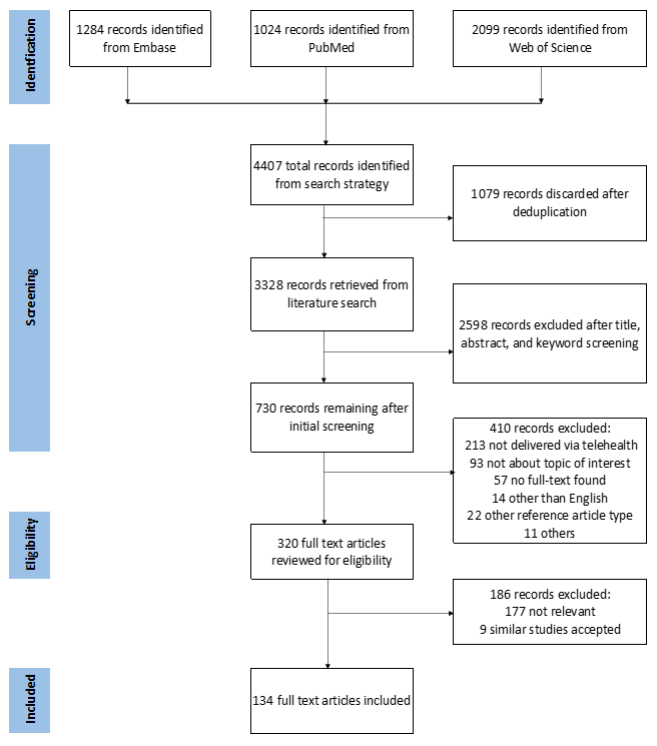
Flowchart for the structured literature search and selection.

Of the 4407 abstracts identified from the search protocol, 134 full-text articles fulfilled the inclusion criteria. A total of 1079 duplicate records were removed, 2598 records were discarded after title, abstract, and keyword search, and 410 records were deemed not fit after the initial screening as these articles were not about topic of interest having objectives that did not align with the outcomes of this review and did not satisfy the inclusion criteria. Of the 320 articles that were subjected to full-text review, 177 articles were deemed not relevant because they either included the digital reality technology or telehealth strategies but not delivered jointly, and 9 were excluded after recognizing multiple papers published on the same topic by the HW, SC, and JK (Figure 2).

### Digital Reality Platform via Telehealth

**Figure 3:**
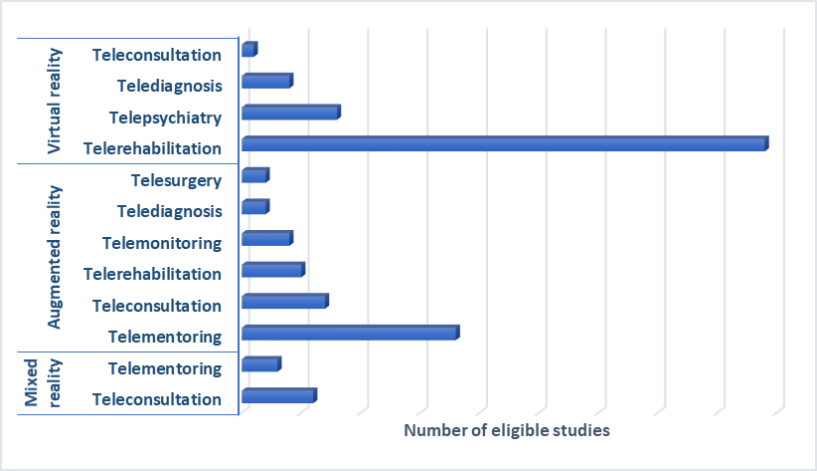
Collaboration between digital reality technology and telehealth for the included studies.

As demonstrated in Figure 3, VR and AR cover most of the listed telehealth domains for the eligible studies. The most studied and researched area is telerehabilitation accomplished using VR. The other subareas involving VR use include telepsychiatry for evaluation and treatment, telediagnosis, and teleconsultation. In addition, AR and MR are prevalent modes of the reality technology platform for telementoring and teleconsultation. Finally, telesurgery and telemonitoring are the 2 subfields of telehealth where AR technology have seen an upward trend.

**Figure 4:**
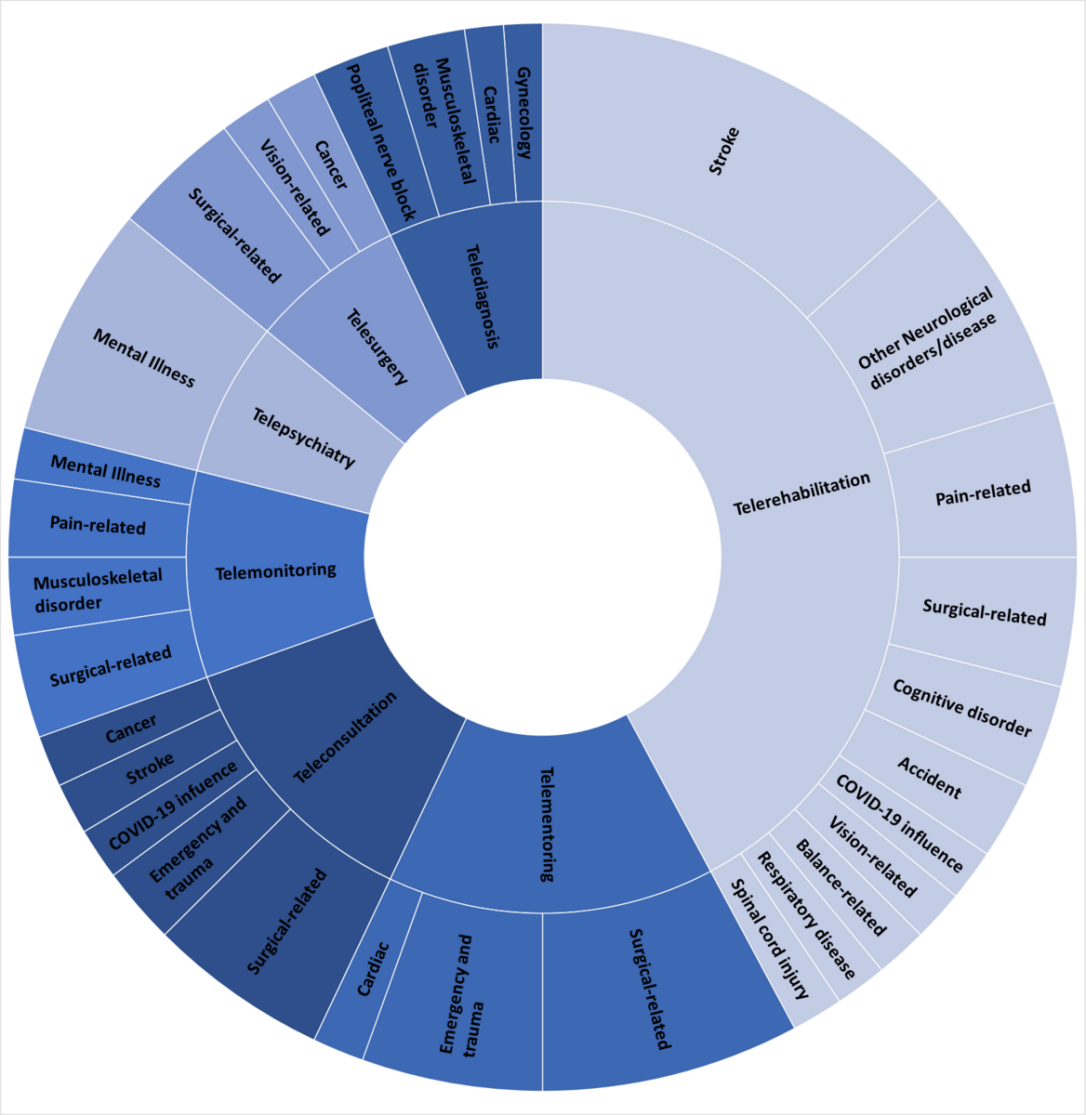
An overview of different clinical conditions and groups categorized within specific telehealth domains.

Clinically based digital health applications were considered for the review as various specific branches of the telehealth spectrum (Figure 4). *Telerehabilitation* is a postclinical care service delivered at home or remotely for recovery purposes and constitutes most of the included telehealth group from the included studies [21,22]. Evident from the included studies, stroke rehabilitation emerges as the leading medical condition that has seen an uptake of these services. Different aspects of rehabilitation, such as functional motor training, including upper-extremity training and fine motor skills, cognitive functional training, visuomotor tracking training, and balance and gait training are primarily used for treating poststroke survivors [23-41]. In turn, the patient groups who have used telehealth for the purposes of rehabilitation have reported improvements in their quality of life, increased daily activities, and improved levels of motivation [42]. From the multiple studies included, telerehabilitation has been experimented as a home-based treatment for various neurological and cognitive disorders or diseases such as Parkinson disease, acquired brain injuries, multiple sclerosis, cerebral palsy, mild cognitive impairment, Alzheimer disease, and dementia [43-58].

Conventional therapy programs in the form of physical therapy and behavioral therapy are the nonpharmacological treatments that have used this remote delivery platform. In a small number of studies, the home-based rehabilitation in the form of novel telerehabilitation have been used for patients undergoing surgical procedures, such as total hip replacement, total knee arthroplasty, and total knee replacement, as a postrecovery treatment measure [59-62]. Mirror therapy for patients with phantom limb pain and physiotherapy treatment for patients with chronic body pain have incorporated this model of remote teletherapy [63-68]. This field has also been applied in physical rehabilitation for musculoskeletal disorders, provision of vestibular rehabilitation therapy in patients with a balance disorder, and kinesiotherapy for older adults at risk of falls [2,69,70]. Physical therapy in the pediatric group and musical therapy in patients with spinal cord injury have explored this stream of technology [71,72]. *Pulmonary rehabilitation therapy* for respiratory disorders such as chronic pulmonary respiratory disorder, pulmonary fibrosis, and myocardial infarction; *low vision rehabilitation* in providing functional visual assistance; and the *COVID-19 pandemic* have been an influential factor in accelerating remote rehabilitation therapy [22,73-77].

*Telementoring* is a subset of telemedicine that reflects remote expert guidance such as training or telenavigation to medical and nonmedical personnel in performance of life-sustaining procedures [78]. The impact and usability of the telementoring technique in provision of cardiopulmonary resuscitation in treating cardiac arrest has been demonstrated by different authors in simulated environments, with the assistance of a remote mentor using an HMD or Google Glass [79,80]. Other authors have explored the use of telementoring guidance, in intraoperative telenavigation, and preoperative planning in simulated battlefield and emergency trauma. The telementoring approach for preoperative planning and telenavigation during the intraoperative process has been demonstrated in complex emergency hand reconstruction surgery [81,82]. Forward damage control procedure performed on a patient-simulator model depicting a right-sided femoral gunshot wound and simulated trauma injuries such as airway obstruction by conducting cricothyroidotomy have been carried out using remote instruction—as have, lung decompression, tracheostomy, or REBOA (resuscitative endovascular balloon occlusion of the aorta) catheter deployment to deal with specific trauma injuries with the aid of a remote medical expert [82-86]. The feasibility of telementoring applicability in the performance of chest thoracotomy, skin grafting, and fasciotomy has been evaluated using ex vivo animal models [78,87,88]. Telementoring has been used to great effect in different stages of surgical planning in various orthopedic, craniofacial, spinal cord, vascular, and cardiothoracic surgeries [6,89-100].

Teleconsultation is a primary segment of telehealth services, broadly consisting of remote consultation services using ICT. This remote consultation can be synchronous or asynchronous and between clinicians (provider-to-provider) for shared decision-making or between clinicians and their patients (provider-patient) [101]. This approach has been applied in patient assessment using the National Institute of Health Stroke Scale for patients with acute stroke and during remote clinical rounds in isolation wards for patients with COVID-19, thereby reducing direct exposure of the staff [102,103]. This technique has also been evaluated in trauma and emergency-related scenarios, such as remote consultation in reading and interpreting electrocardiogram reports related to drug intoxication or poisoning [104,105]. The effectiveness of provider-to-provider teleconsultation has been demonstrated in provision of support for ambulatory staff and first-responders in triage during simulation of major trauma [106]. The applications of teleconsultation in provision of surgical care are broad, allowing collaborative, contextual, and presurgical planning and visualization and intraoperative surgical navigation through high-fidelity immersive reality platforms and devices, as well as facilitating remote delivery of complex information to patients [107-115]. Teleconsultation via the reality platforms has been used to explore the feasibility of telepathology in carrying out an autopsy, image scanning, and transfer of serially sectioned cancer tissue from a mouse [116].

*Telemonitoring* is an advanced form of clinical care service that provides patient-centered care. This method allows health care providers to collect and track patient information and deliver remote care assistance [117]. This branch of telehealth has been evaluated in pediatric cohorts dealing with hospital-induced stress as a shared experience on a mobile-based AR game for play therapy. This aspect allows managing pediatric patient profiles, data collection, and further analysis for effective treatment [118]. Telemonitoring via holographic conversational agents; that is, a computer-generated character to deliver physiotherapy home exercises to patients with musculoskeletal disorders and chronic pain has been demonstrated to increase their treatment adherence [119]. Supervised AR-based home training has been used for patients with phantom limb pain by providing mirror therapy, thereby promoting visuomotor integration by reengaging the neural circuits related to lost limbs [120]. Telemonitoring has been used in postoperative care and wound assessment in orthopedic and neurosurgical cases and has also been applied for *teleproctoring* or *remote monitoring* in pilot simulation as training for fundamentals of laparoscopic surgery examination [121-123].

*Telepsychiatry* uses ICT to offer a range of clinical and nonclinical services such as psychiatric evaluation, therapy (individual or group-based), patient education, and management remotely [124]. Studies using this element of telehealth and computer-generated virtual environments have evaluated the feasibility of remote therapy such as Virtual Reality Exposure Therapy for patients with acrophobia and evaluation of the technical system in delivering specific phobia treatment for arachnophobia [125,126]. Remotely delivered psychological treatment by the mental health professional include behavioral intervention therapy, cognitive behavioral therapy, mindfulness therapy, and acceptance and commitment therapy for patients facing stress, anxiety, public speaking anxiety, and social anxiety disorder, among others [127-130]. In a simulation study, cognitive and affective assessment of astronauts has been carried out to characterize social isolation from space [131]. Evaluation of telepsychiatry using the reality platform such as VR versus the traditional videoconferencing platform, and the development of newer platforms such as social VR for older adults in urban areas has demonstrated such techniques could lead to improved quality of life by reducing social isolation [132]. Telepsychiatry assessment via VR as a home-based treatment delivered by mental health professionals, such as a psychiatrist, psychologist, licensed social worker, or a mental health counselor, has been demonstrated to mitigate clinician burnout [133,134].

Another exciting subsection of the telehealth sphere, *telesurgery*, enables teleoperation in an operating field executed over a distance. Telesurgery involves using various disciplines such as communication technology, imaging techniques, motor control systems, robotics, reality platforms, and digital signal processing [135,136]. For example, in an experimental setup, a VR-based teleoperative system consisting of a robotic catheter operating system can be used to imitate vascular interventional surgery for arterial aneurysms or other vascular diseases. This method allowed unskilled surgeons to train in essential catheter guidance skills and enabled experienced physicians to conduct surgeries cooperatively [137]. In addition, a telesurgical experiment was conducted with a tendon-driven continuum robot via telenavigation for endoscopic and minimally invasive surgical procedures by tracking coordinate trajectory registration [138]. Finally, in another simulation case, a magnetically driven endoscopic capsule enabled the teleoperator or user to receive visual feedback in VR to conduct capsule endoscopy for colorectal cancer [139].

Moreover, the reality platform is streamed as a functional stereoscopic display and navigates space during telesurgery. This aspect of telesurgery has been experimented with as a visualization opportunity using smartphone-delivered vision and VR headsets to perform microsurgery for cataract and phacoemulsification [140]. In addition, Stereoscopic AR Predictive Display using the da Vinci R Surgical System to perform laparoscopic surgery and AR-assisted robotic surgery for kidney transplant procedures are some of the current practical applications of telesurgery [141-143]. *Telediagnosis* refers to the detection or evaluation of a disease or condition using telematics technology. It is achieved remotely while the patient is at a local site with remote diagnostic tools and devices [144,145]. For instance, in experimental analysis, locating and evaluating tumor-bearing hysteromyoma coordinates during a 3D navigated gynecological operation facilitates telediagnosis when visualized on a 3D user interface of the medical record [146]. Another study proposes a framework based on bidirectional haptic feedback and tele immersion in the evaluation of range of motion and maximum isometric strength using the 10 arm movements method in the diagnosis of musculoskeletal disorders, poststroke rehabilitation, or postshoulder surgery [147]. Ultrasonography (USG) is a field in which telediagnosis using the high-fidelity visualization system has been used to great effect. Evaluation of 3D VR telenavigation in cardiac USG has been undertaken in simulated settings. The added benefits of AR enable real-time teleguidance on procedural performance and image registration for point-of-focus ultrasonography (POCUS) and foveated imaging pipeline in extending VR-based telediagnosis [148-150]. Another study mentions AR video communication projected by mobile-based AR guidance to conduct POCUS on popliteal nerve block and a subsequent diagnosis based on the availed health information [151].

### Overview of the Hardware and Software Units for the Included Studies

To experience MR, high-simulation visualization hardware devices and some of the commercial ones included in the selected studies are listed in Multimedia Appendix 2. These include high-end AR and VR devices, smart glasses, mobile devices, standard LED (light emitting diode) and LCD (liquid crystal display) television or display screen, 3D television, and 3D projectors. The commonly included immersive reality-capable devices are mostly wearable technology such as smart glasses, VR or AR HMDs, and nonimmersive standard display units. However, these high-fidelity simulation display technologies form the final part of any system and are primarily used in combination with optical capturing and tracking devices and input devices. The optical capturing and tracking systems or devices incorporate 3D depth and color-sensing camera sensors. The input devices such as controllers, trackers, or customized input modules help navigate the immediate VR or any MR environment. Various studies have included the VR gaming element in their rehabilitation programs, with some having their own developed VR rehabilitation system. Most included studies have used biometric devices for specific medical parameter evaluation to draw analysis and simulation models to conduct various training. Other relative hardware devices and systems that have been used are listed in the Multimedia Appendix 3.

The graphical representation for any software harbors a visualization platform, and more specifically, the MR system incorporates contextual 3D figures and scenes. The various software applications and source platforms that were featured and used in the included studies are listed as a table in the Multimedia Appendix 4. These applications are grouped as 3D modeling and visualization software, communication and streaming software, file-sharing and transfer applications, and other specific and personalized software applications. The 3D composite images and environment for the MR technology are created using the computer graphic designing software and gaming engine platforms. Processing and accessing the 3D computer-generated environment or images needs specific and compatible visualization file applications supported by the device. The featured 3D modeling and viewing application allows for creating and editing static and interactive multidimensional models and VR scenes, animations, and games, conversion of the produced scanned images to computer-aided design models, and stereoscopic 3D display content. The telehealth domain explores the ICT for effective remote clinical services while using the streaming facilities offered by various low-bandwidth platforms. This domain allows offline or real-time interactive communication and collaboration for any dedicated clinical services. Many communication and streaming applications allow for remote one-to-one or group video calls and messaging, screen sharing, file sharing, hosting channels, and video broadcasting. Some of these platforms allow for direct AR and VR integration and acceptance. The file-sharing and other specific applications are synchronously and explicitly used as a sequential fragment of the entire system. The developed software from the studies mentioned in the table encapsulates the combination of AR and VR cooperatively with the remote telehealth applications.

### Virtual Environment

A virtual environment (VE) recreates a coordinated appearance of sensory information representing that of a physical environment that can be unreal, interactive, or wholly imagined environment perceived when the user wears an appropriate gadget [152]. In addition, the term *virtual* *worlds* has been interchangeably used with a VE. Developing this state-of-the-art perceived environment is created using a subset of tools arising from computer game technology, specifically through commercial game engines. The scene can be a 2D or 3D illustration, which is a complex and time-consuming process for its creation [153]. This element of VE has been recreated in almost every aspect using the VR platform. For example, the study by Levy et al [125] demonstrated the use of virtual worlds such as a subway station and a 24-story high-rise building as background scenes to overcome acrophobia as a VR exposure therapy. Similarly, Cikajlo et al [127] developed a program called ReCoVR (Realizing Collaborative Virtual Reality for Well-being and Self-Healing). The participant attends a remote guided mindfulness program as part of a group. This mindfulness program was organized as 360° video scenes where they carried out different tasks and exercises. Initially, all the participants that joined were seated in the virtual fireplace room; upon the program’s progression, they were switched to other 3D VEs, such as the Dooney Rock, River Bonnet, or the mountain-view. Shao and Lee [132] have addressed a social VR platform that uses the 3D scenes in the VE for real-time face-to-face communication in different distant locations to learn about its value and urgency in the urban older adult population. Tamplin et al [72] developed a web-based music therapy telehealth platform using social VR, vTIME (vTime Limited), allowing group music therapy sessions in VE, such as singing around a campfire in a forest.

### Gaming-Based VE

Moreover, many studies used VE in interactive game–based settings for rehabilitative exercise programs. In a program described by Meca-Lallana et al [53], patients were required to carry out specific tasks to accomplish a mission in 2 different scenes: a medieval fantasy world and a deserted island. Yet again, in another exercise setup, VR exercises depict a wooden church in Hrabova Roztoka. The patient explores this particular place using a VR headset, thereby facilitating lower-limb rehabilitation [36]. Telerehab VR, a custom-built application program that runs on either a mobile-based tablet or PC, was developed using the game engine Unity (Unity Technologies Inc). This system provides upper-limb rehabilitation for patients with multiple sclerosis. They perform various activities of daily living tasks happening in the VE in a realistic home setting. A leap motion controller (Ultraleap) was used to track and control the hand motion executed while performing the gaming tasks [48].

### Telepresence

*Telepresence* describes the characteristic of directly interacting with the actual physical state, experienced from the first-person viewpoint of the user located remotely [154]. Tian et al [147] used the H-TIME (Haptic Enable Tele-Immersion Musculoskeletal Examination) set up at both the patient and doctor ends to conduct a remote diagnosis of musculoskeletal examination. At both sites, the doctor and patient could feel each other’s movements because of the bidirectional force feedback mechanism. They could view and communicate with each other in the VE, bringing them to the same examination room virtually. In another instance, in treating phobia, in particular, fear of spiders, the patients were allowed to interact in the VE, where the therapist gradually added the feared creature to the scene. This treatment is performed remotely via the tactile internet with VR headsets or standard computer screens using a hand-tracking and haptic device such as a glove [126].

*Teleoperation* refers to performing designated highly skilled manual tasks remotely, similar to a telerobotic medical system in minimally invasive surgery [155]. In a simulated study, an endoscopist performs a teleoperation process using a haptic device that controls the position of an external permanent magnet positioned at the end of a robotic arm. The user is wearing a VR headset and receives the corresponding visual information from the camera of the endoscopic capsule and then proceeds with the navigation process inside the colon [139]. Another simulated study used teleoperated ultrasonography that builds on the VE developed as a 3D representation of a real USG probe and a mannequin imitating a patient’s body highlighted with a geometric mesh for the purpose of following the examination. The user wears an Oculus Rift DK2 HMD (Oculus) to perform this simulation of tele-USG [148]. Syawaludin et al [150] introduced the use of 360° foveated pipeline imaging viewed via an HMD. The image or video capture is facilitated by the use of an omnidirectional pantilt-zoom camera module, and the remote physician can remotely diagnose the wound by zooming in and inspecting it in a 360^◦^ view over the HMD.

### Exergaming and Serious Gaming via VR

In the context of virtual telerehabilitation, *exergaming* and *serious* *games* are the 2 most popular applications that emerge. Simply put, exergaming is an activity connected with playing video games that involve physical exercise [70]. In contrast, serious games follow the objective of games, implicitly focusing on increasing skills and abilities and gaining experience and knowledge [156]. The TELEKIN system, a beta edition, uses the interface of the serious game to rehabilitate cognitive and musculoskeletal disorders using a web-based framework [43]. The training sessions are conducted and played in a 3D VE that includes a number of games. Two of them involve physical actions—flexion and extension of wrist, hip, or shoulder as the doctor chooses to control the ball and platform in executing the game. Another game focuses on cognitive rehabilitation by featuring random words that the user must arrange them to form a sentence, which can be achieved using gesture-based controls [43]. Gandolfi et al [44] used the Tele Wii Lab platform as a home-based balance training, and Sheehy et al [28] and Allegue et al [30] used the Jintronix system (Jintronix, Inc) in upper-extremity rehabilitation of chronic poststroke patients, which used the exergaming platform. The study by Triandafilou et al [24] that developed a networked multiuser gaming format, Virtual Environment for Rehabilitative Gaming Exercise (VERGE), conducted a feasibility trial to determine the effectiveness of this developed system with other potential home treatments. The VERGE system features a set of 3 exercises, namely Ball Bump, where the users pass the ball back and forth across the table; Food Fight, where the users in multiplayer can pick up the food on the table and throw it at each other; and the Trajectory Trace game, where 1 player draws a trajectory path in the space while another player retraces the trajectory to erase it. Burdea et al [32] included a commercial rehabilitation system with a novel therapeutic game controller, BrightBrainer (Bright Cloud International Corp). This system offers a multitude of interactive games (Breakout 3D and Card Island Towers of Hanoi, among others), training motor, cognitive, and executive functions for chronic poststroke patients. Qiu et al [35] demonstrated the feasibility of a home-based VR system that features 12 developed games focusing on the elbow-shoulder, hand, wrist, and entire arm for upper-extremity rehabilitation in poststroke patients (finger games: car, bowling, and piano; hand games: piano and fruit picking; wrist games: Wakamole and wrist flying; and finally, the shoulder-elbow games: the Maze, Arm Flying, Brick Break, and soccer goalie)

### Avatar Representations or Virtual Agents

The term *avatar* is a distinguishable digital characterization of a human form (either specific or random) [128]. Moreover, these avatars can be either in 2D or 3D illustration, representing a specific part of the body, usually arms or an entire body structure with particular facial expressions. 3D avatars have been a central representation in the scope of VR and AR. The study by Anton et al [59] implements the Kinect-based Telerehabilitation (ie, KiRES) interface, providing two 3D avatars to guide the patient during their physical therapy session. One of the avatars represents the remote therapist and represents the local user or the patient, colored red and blue, respectively, so the patient can follow and perform the exercises executed by the 3D remote therapist avatar (in red). The patient can see their movements reflected by their blue avatar changing their positions as per the scenes from the therapy. In the study trial conducted by Jung et al [74], in a telerehabilitation program—Pulmonary Rehabilitation in Virtual Reality (PR in VR) program—each patient was provided with a VR headset, pico G2 4k (Pico Immersive Pte Ltd), preloaded with the PR in VR application. This application contains education and rehabilitation modules, and the chronic obstructive pulmonary disorder rehabilitation module comprises several physical exercises directed by a virtual instructor in 3D avatar embodiment.

The REWIRE autonomous telerehabilitation platform offers home-based intensive rehabilitation as offline remote monitoring by hospital clinicians. This system features a virtual therapist with artificial intelligence implanted and provides real-time feedback to maintain correct posture. In addition, the exercises performed by the patients are showcased as a 3D avatar on the screen. This intelligent system highlights each body segment of the exercise in a different color, intense green for the proper posture and red color for the incorrect posture [23]. The VERGE system enables the use of avatars to control and manipulate objects in the virtual gaming environment, allowing the capability to include multiple avatars and different users to manipulate the same object [24]. In the social VR app, vTIME (vTime Limited), an avatar persona is used for self-characterization to communicate in an immersive VE [128]. Afyouni et al [65] describe the use of RehaBot, a virtual assistant that illustrates to the user how to perform the exercises correctly (both the therapist and patient can replay the session in a 3D avatar). The RehaBot embeds real-time pattern and gesture recognition together with a dynamic correction module that considers the game difficulty level and reading from the virtual assistant to produce a tailored set of exercises that are rather fitting to the patient’s native abilities.

### Telestration and Annotations via AR and MR

*Telestration* enables the drawing of freehand representations, also known as annotations (such as lines, circles, or any other symbols or sketches) over any image or video feed. With the latest AR and MR technology, this telestration can be achieved in 2D and 3D and superimpose this annotation in the live video streaming during the video call [157]. The Virtual Interactive Presence and AR tool is a mobile or tablet-based augmented reality platform running on an iPad device (Apple). It incorporates the telestration feature, allowing the remote expert surgeon to freeze the screen and then draw an image using a 2D pen tool. This composite video feed, viewed on both the local and remote stations, enables intraoperative telecollaboration in real-time [90]. In the feasibility study by Wang et al [100], POCUS using the HoloLens was conducted by the trainee in a simulated teleconference session. The MR capture video from the trainee was broadcasted. Live guidance provided by the expert mentor facilitates the trainee to complete a right quadrant Focussed Assessment using Sonography in Trauma examination. The broadcasting was achieved using VSee, a proprietary low-bandwidth, group videoconferencing and screen-sharing application. To perform complex hand reconstruction of a patient after a bomb-blast injury, a telementoring network was established between an expert surgeon in Lebanon and a local surgeon in Gaza. This session was hosted using a cloud-based AR platform Proximie (Proximie Limited), allowing the remote surgeon to superimpose their own hands or range of annotations and drawing tools into the virtual surgical field [82].

Mitsuno et al [93] demonstrated telementoring in a simulation study to perform craniofacial surgery by using a teleconferencing setup, Skype (Microsoft Corp) for HoloLens, enabling the telestrated features and images overlaid on the receivers’ visual field. A POCUS examination was performed using a novel smartphone app Vuforia Chalk (PTC Inc), an AR video platform for remote AR assistance, anchoring the AR annotations in each other’s supposed visual environment [151]. In the study, Ritcher et al [141] proposed the first predictive display with AR registration and rendering using stereoscopic displays designed for teleoperated surgical robots known as Stereoscopic AR Predictive Display. The simulation study measured the effectiveness of Stereoscopic AR Predictive Display conducted on the da Vinci R Surgical System (Intuitive Surgical) to complete the peg transfer task. The System for Telementoring with AR (STAR) platform now combines optical see-through display, HoloLens AR HMD. Similarly, this system allows for telementoring guidance by overlaying 3D graphical annotations onto the mentee’s view of the surgical field, which remains anchored in the same place even after the mentee moves their head position [95].

The experiment by Zhang et al [110] aimed to enhance teleconsultation by using the AR technology ARkit (Apple) to create an immersive replica of the consultant. Using a Kinect sensor (Microsoft Corp) to capture the skeletal feature points of the consultant, the patient views a 3D dynamic virtual avatar doctor appearing in the patient’s telepresence environment on their iPad device. A qualitative study was conducted to gain the experience and perception of AR Glasses in patients with pulmonary disorders for home-based telerehabilitation. The web-based telerehabilitation system Optimov (Optimov) enabled via an AR Glasses device Laster WAVƎ headset (Laster Technologies) provides exercise coaching using a 3D virtual agent [73]. A holographic virtual therapist was deployed in the HOLOBALANCE, a novel health care platform for providing vestibular rehabilitation therapy for patients with balance disorders [158]. In the design and evaluation user study by Kowatsch et al [119], a hybrid ubiquitous coaching model relying on mobile and holographic conversation agents was introduced. The 3D virtual conversation agent demonstrated the squat exercise, engaging in real-time audio feedback for counting the repetition or providing automatic error detection for incorrectly or incompletely following the exercise. An innovative 3D point tracking module and unique AR system integrated with the HoloLens was used for surgical applications using telementoring. This module allowed for real-time 3D position tracking of the virtual scalpel handled by an experienced surgeon remotely. The inexperienced trainee wearing the HoloLens can see the surgical annotation superimposed with the actual surgical scene; the virtual path coregistered on the phantom arm model [78]. Next-generation mobile-based AR games for pediatric health care allow shared experiences with multiple other AR-supported devices to detect and interact using the same local area network. Several games were developed using the Unity game engine and ARCore Unity, a software development kit for Android operating software. Jungle Adventure, Map explorer, and Wakamole implemented AR interaction, whereas Map explorer and Wakamole particularly enabled the inclusion of a 2-player for a shared collaborative experience [118].

### First-Person View for AR Capture Video Feed

Noorian et al [102] demonstrated smart reality glasses to conduct remote consultation using the National Institutes of Health Stroke Scale scores for stroke assessment. The onsite doctor wears the reality glass, Google glass. This Google glass is embedded with the Xpert Eye platform (AMA XpertEye), capable of assisted reality, allowing the person wearing this device to share their field of view in a 2-way real-time videoconferencing. Similarly, Nikouline et al [123] presented a feasibility study using the Google glass live video stream coming from the onsite proctor and the participant tasks related to fundamentals of Laparoscopy for scoring and evaluation done by the remote proctor. In their experiment, Lin et al [84] implement projective video texture-mapping that supplements a robust high-level stabilization video feed obtained from the mentee’s first-person view. This effective format provides the remote expert with an effective workspace visualization, allowing seamless integration of annotations in an effective AR surgical telementoring. The prospective observational study by Martin et al [103] uses HoloLens 2 MR device to conduct remote clinical consultation in a COVID-19 ward. A senior staff member would enter the COVID-19 ward to undertake clinical rounds, and the other staff members of the staff team would join virtually, thereby minimizing exposure and infection transmission. Dynamic 365 Remote Assist (Microsoft Corp) software allowed for bidirectional audio and video functionality through which the remote staff team could see the first-person view from the HoloLens 2 device. In addition, this platform allowed to place relevant imaging and electronic health record data in the user field of view, improving situational awareness and better clinical decision-making. Finally, it significantly reduced the risk of direct viral transmission.

### Web- and Cloud-Based Telehealth Delivery Modes

As digital communication network and services evolve, these are rapidly being adopted in health care delivery. The web- and cloud-based applications have become prevalent in telehealth. Telehealth relies on the backbone of internet infrastructure supported using various broadband connections such as digital subscriber lines, fiber broadband, and wireless connection, including fixed wireless broadband, cellular network or mobile broadband, and satellite communication. Thus, ICT has become central to offering an array of digital health solutions such as real-time audio and videoconferencing, remote patient monitoring, store and forward technologies, and mobile health, among others [159,160]. A web-based application principally operates on the webserver. It is accessed through a web browser over an internet connection, whereas cloud-based applications operate similarly to web applications, operating on either or both the client and server sides [161]. The custom-developed systems KiRES [59] and STAR [85] rely on the WebRTC framework, an open-source application programing interface allowing for real-time audio-video and multimedia connection. In addition, the study by da Silva et al [55], included a web-based gaming application MoveHero, to evaluate the feasibility of home-based nonimmersive serious games in patients with cerebral palsy.

Interestingly, any virtual web-based application feeds on the information; thus, data storage and hosting become integral to all online services. The study by Kato et al [42], adopted the cloud-based storage and file hosting service Dropbox (Dropbox Inc) for collecting the spatial coordinate data for each joint using the 3D optical camera during the VR telerehabilitation. The proof-of-concept study by Sirilak et al [107], implemented an e-consultation system based on the AR and MR systems using the HoloLens device for remote consultancy services in the intensive care environment. This e-consultation platform depended on a cloud-based data center that performed as an information exchange and provided services for the end devices. It also consisted of body area network technology to integrate the vital physiological information from different client devices to the data center. Prvu Bettger et al [62], used a virtual telehealth system—virtual exercise rehabilitation assistant or virtual exercise rehabilitation assistant (Reflexion Health, Inc)—for posthospital care for total knee arthroplasty, Tsiouris et al [2] included a custom-developed platform HOLOBALANCE system in managing balance disorders, both using the technology-forward cloud-based platform.

## Discussion

### Principal Findings

This scoping review explores state-of-the-art extended reality platforms and telehealth solutions used in the clinical context. This review highlights the reported evidence-based practical and probable applications of the extended and MR platform with telehealth used in different clinical specialties. This review also addresses the technical characteristics of the AR and VR features used in telehealth services, including various hardware and software arrangements.

Stroke is the leading clinical condition incorporating telerehabilitation, a segment of the telehealth service and digital VR [23-41]. Approximately half of the included studies from the search strategy feature the use of telerehabilitation (Figure 4). Other clinical conditions such as neurological or cognitive disorders, musculoskeletal conditions, and postsurgical recovery have also adopted telerehabilitation facilities in the home or remote settings to continue treatment. Telerehabilitation used technical attributes of exergaming and serious gaming in improving the motor and cognitive functional skills [43-58].

Other divisions of telehealth, namely telementoring, teleconsultation, and telemonitoring, have been more frequently used for surgical-related procedures, emergencies and trauma, and in several disaster simulation for disaster response and preparedness [162]. The AR and MR technologies are more prevalent with telementoring, teleconsultation, and telesurgery (Figure 3). Exposure therapy under telepsychiatry has used VR to give the patient a photorealistic experience of overcoming their pathological response to their fear [126].

Telestrated AR features through anchoring of annotations in real-time and space, performed remotely via various communication channels: a useful aid in telesurgery [81]. The technical features from the digital reality technology of VE, digital avatars, telestration or the 3D rendering of annotations, and first-person viewpoint have demonstrated telemedical capabilities. The web- and cloud-based applications have various potential uses across the web-based clinical sphere [110]. Most of the included studies relied on existing commercial high-fidelity simulation technological hardware devices such as head-mounted AR and VR displays. The study and software designs for most of the included studies were codeveloped by the respective research teams by using multiple supportive platforms as a direct requirement for the project objectives.

These novel reality technologies of AR, VR, and MR enable 3D visualization, thereby creating a visual sense and experience of high ecological validity [163]. This technology has been extended as a remote, home-based solution for patients, thereby enabling patient empowerment [164]. This technology is highly engaging and motivating from the patient responses to telerehabilitation, consequently necessitating initial patient training needs may become an arduous task to the facilitator [42,165]. Network connectivity, internet and server security concerns, and technological constraints are some of the most common pitfalls across several studies included in this review [99,166]. The lack of interoperability between the hardware and software platforms poses a significant challenge in realizing the potential of this technology [2]. The need for improved network infrastructure and scalability poses a challenge and target for telehealth services; however, there is a risk of complete network failure, which can affect the use of such systems in critical care applications [83]. Patient confidentiality is integral at any stage during electronic exchange of health-related data; thus, network and data security protection are crucial factors for accessing telehealth services and should be robustly adhered to the governing regulations [66,149,167].

The exploded search strategy captures a broad array of important clinical applications of this high-fidelity reality technology and telehealth facilities. This review presents a current road map and the prospects of digital reality technology and telehealth in the clinical space. The determining factors presented allow the readers and researchers to evaluate the relevance of this technology and its subsequent uptake in the clinical health sector. The study protocol was not registered, the included studies were not classified for risk of bias assessment, and the general characterization for the included studies were not presented. In addition, the review only included studies available in the English language and no relevant additional pieces of information was considered from the gray literature. From the broad array of literature-based evidence, most of the included experimental studies were pilot feasibility studies with small sample sizes, leading to reporting bias.

### Conclusions

This review uniquely details the current and potential applications of digital reality technologies such as VR and AR and telehealth solutions. The feasible and practical application of AR and VR in the digital clinical space has been explored, as well as the challenges this multiparty technology endures in effective implementation and adoption. This suite of technologies offers a collaborative experience among health care professionals and their patient community. The telehealth component with the high-fidelity digital reality allows for an immersive and integrative means for teleconsultation, telesurgical procedures, and telementoring among the medical peer-to-peer group allowing for effective decision-making and treatment approaches. The uptake of VR and exergaming in various telerehabilitation programs has opened new avenues to posttreatment measures. This essential application of telehealth enhances the traditional health care delivery approach by enabling remotely delivered clinical care and services and developing home-based treatment programs. Further validated studies are needed to evaluate the overall assessment of this trending technology, thereby leading to commercial pathways. A robust and secure communication infrastructure will improve the accessibility of telehealth capabilities and extend the interoperability of the digital reality platform allowing for a diverse digital health care ecosystem.

## Appendix

Multimedia Appendix 1 Search strategy.

Multimedia Appendix 2 Augmented, virtual, and mixed reality hardware devices.

Multimedia Appendix 3 Other relative hardware devices.

Multimedia Appendix 4 List of software and source platforms.

## Abbreviations

AR: augmented reality
HMD: head-mounted display
H-TIME: Haptic Enable Tele-Immersion Musculoskeletal Examination
ICT: information and communication technology
LCD: liquid crystal display
LED: light emitting diode
MR: mixed reality
POCUS: point-of-focus ultrasonography
PRISMA-ScR: Preferred Reporting Items for Systematic Reviews and Meta-Analyses Extension for Scoping Reviews
REBOA: resuscitative endovascular balloon occlusion of the aorta
ReCoVR: Realizing Collaborative virtual reality for well-being and self-healing
STAR: System for Telementoring with Augmented Reality
USG: ultrasonography
VE: virtual environment
VERGE: Virtual Environment for Rehabilitative Gaming Exercise
VR: virtual reality
