# Supplementary material for: Mixed Reality Platforms in Telehealth Delivery: Scoping Review
Source: JMIR Biomed Eng. 2023 Mar 24;8:e42709. doi: 10.2196/42709 (PMC11041465; doi:10.2196/42709)
Supplement: Multimedia Appendix 1 [file biomedeng_v8i1e42709_app1.docx]

**Multimedia Appendix 1**

**Headings: Telemedicine; Telehealth; Digital realities**

**Keywords: Augmented Reality, Virtual Reality, Mixed Realities, virtual reality headsets, AR headsets, e-health, m-health.**

**Search Strategy – Database**

| \| **Search No.** \| **Query** \| **Results** \| \| --- \| --- \| --- \| \|  \|  \|  \| \| **Embase** \| \| \| \| #28 \| #27 AND (2010:py OR 2011:py OR 2012:py OR 2013:py OR 2014:py OR 2015:py OR 2016:py OR 2017:py OR 2018:py OR 2019:py OR 2020:py OR 2021:py) \| 2120 \| \| #27 \| #19 AND #26 \| 2974 \| \| #26 \| #20 OR #21 OR #22 OR #23 OR #24 OR #25 \| 475379 \| \| #25 \| 'mhealth' OR 'mhealth'/exp OR 'm-health' \| 11864 \| \| #24 \| 'digital health' OR 'ehealth' OR 'e-health' OR 'ehealth'/exp OR 'e-health'/exp \| 70934 \| \| #23 \| 'telemetry' OR 'tele-metry*' OR 'telemetry'/exp OR 'telemetry*':ab,ti,kw \| 35467 \| \| #22 \| 'telemedicine' OR 'tele-medicine' OR 'telemedicine'/exp OR 'telemedicine*':ab,ti,kw \| 59187 \| \| #21 \| 'telehealth' OR 'tele-health' OR 'telehealth'/exp OR 'telehealth*':ab,ti,kw \| 60312 \| \| #20 \| 'tele*' OR 'tele-' \| 445686 \| \| #19 \| #5 OR #10 OR #11 OR #12 OR #13 OR #18 \| 31887 \| \| #18 \| #14 OR #15 OR #16 OR #17 \| 155 \| \| #17 \| 'ar/mr' \| 74 \| \| #16 \| 'ar/vr' \| 32 \| \| #15 \| 'ar and vr' \| 31 \| \| #14 \| 'augment*' AND 'virtual*' AND 'mix*' AND 'extend*' \| 23 \| \| #13 \| 'extended reality' OR 'extended-reality' OR ('reality' NEAR/1 'extend*') \| 55 \| \| #12 \| 'mixed reality' OR 'mixed-reality' OR 'mixed reality':ab,ti,kw OR 'merged-reality' OR 'merged reality' \| 673 \| \| #11 \| 'augmented virtuality' OR 'augmented-virtuality' \| 13 \| \| #10 \| #6 OR #7 OR #8 OR #9 \| 29346 \| \| #9 \| 'reality' AND 'virtual*' \| 27614 \| \| #8 \| 'virtual reality head mounted display' OR 'virtual reality display*' OR 'virtual reality devices*' OR 'virtual reality glass*' OR 'vr-headset*' \| 529 \| \| #7 \| 'immersive virtual reality' OR 'immersive*' OR 'immersive reality' OR 'immersive near/1 reality' \| 3480 \| \| #6 \| 'virtual reality' OR 'virtual reality system' OR 'virtual reality system'/exp OR 'virtual reality system':ab,ti,kw \| 26365 \| \| #5 \| #1 OR #2 OR #3 OR #4 \| 4495 \| \| #4 \| 'reality' AND 'augment*' \| 4268 \| \| #3 \| 'smart glasses' OR 'ar headsets' OR 'hololens' OR 'augmented reality display' OR 'augmented reality display devices' \| 544 \| \| #2 \| 'augmented reality system' OR 'augmented reality system'/exp OR 'augmented reality system':ab,ti,kw \| 391 \| \| #1 \| 'augmented reality' OR 'augmented-reality' OR 'augment* near/1 reality*' \| 3378 \| \|  \|  \|  \| \| **Pubmed** \| \| \| \| 12 \| ((((augmented reality OR augmented-reality OR augment* realit*) OR (virtual reality OR virtual-reality OR immersive reality OR virtual* realit*)) OR (augmented virtuality OR merged reality OR mixed reality OR extended reality OR augment* virtual* OR merged realit* OR mixed realit* OR extended realit*)) OR (smart glasses OR heads up display* OR holoLens OR virtual reality headsets OR head mount* display* OR HMDS)) AND ((((tele* OR tele-*) OR (telehealth OR tele-health)) OR (telemedicine OR tele-medicine OR telemetry)) OR (digital health OR ehealth OR e-health OR mhealth OR m-health)) \| 1,165 \| \| 11 \| ((((augmented reality OR augmented-reality OR augment* realit*) OR (virtual reality OR virtual-reality OR immersive reality OR virtual* realit*)) OR (augmented virtuality OR merged reality OR mixed reality OR extended reality OR augment* virtual* OR merged realit* OR mixed realit* OR extended realit*)) OR (smart glasses OR heads up display* OR holoLens OR virtual reality headsets OR head mount* display* OR HMDS)) AND ((((tele* OR tele-*) OR (telehealth OR tele-health)) OR (telemedicine OR tele-medicine OR telemetry)) OR (digital health OR ehealth OR e-health OR mhealth OR m-health)) \| 1,536 \| \| 10 \| (((tele* OR tele-*) OR (telehealth OR tele-health)) OR (telemedicine OR tele-medicine OR telemetry)) OR (digital health OR ehealth OR e-health OR mhealth OR m-health) \| 3,09,772 \| \| 9 \| digital health OR ehealth OR e-health OR mhealth OR m-health \| 89,412 \| \| 8 \| telemedicine OR tele-medicine OR telemetry \| 60,529 \| \| 7 \| telehealth OR tele-health \| 45,839 \| \| 6 \| tele* OR tele-* \| 2,61,178 \| \| 5 \| (((augmented reality OR augmented-reality OR augment* realit*) OR (virtual reality OR virtual-reality OR immersive reality OR virtual* realit*)) OR (augmented virtuality OR merged reality OR mixed reality OR extended reality OR augment* virtual* OR merged realit* OR mixed realit* OR extended realit*)) OR (smart glasses OR heads up display* OR holoLens OR virtual reality headsets OR head mount* display* OR HMDS) \| 22,586 \| \| 4 \| smart glasses OR heads up display* OR holoLens OR virtual reality headsets OR head mount* display* OR HMDS \| 4,071 \| \| 3 \| augmented virtuality OR merged reality OR mixed reality OR extended reality OR augment* virtual* OR merged realit* OR mixed realit* OR extended realit* \| 7,389 \| \| 2 \| virtual reality OR virtual-reality OR immersive reality OR virtual* realit* \| 14,700 \| \| 1 \| augmented reality OR augmented-reality OR augment* realit* \| 3,366 \| \|  \|  \|  \| \| **Web of Science** \| \| \| \| # 16 \| #14 AND #9 \| 3,139 \| \|  \| Refined by: PUBLICATION YEARS: ( 2021 OR 2013 OR 2020 OR 2012 OR 2019 OR 2011 OR 2018 OR 2010 OR 2017 OR 2016 OR 2015 OR 2014 ) \|  \| \| # 15 \| #14 AND #9 \| 4,788 \| \| # 14 \| #13 OR #12 OR #11 OR #10 \| 5,77,409 \| \| # 13 \| TS=(digital health OR mhealth OR m-health OR ehealth OR e-health) \| 42,865 \| \| # 12 \| TS=(telemedicine OR tele-medicine OR telemetry OR tele-metry) \| 50,426 \| \| # 11 \| TS=(telehealth OR tele-health) \| 10,162 \| \| # 10 \| TS=(tele*) \| 5,40,652 \| \| # 9 \| #8 OR #7 OR #6 OR #5 OR #4 OR #3 OR #2 OR #1 \| 1,00,697 \| \| # 8 \| TS=(smart glasses OR head mount* display* OR head mount* OR HMD* devices* OR heads up display* OR heads up device* OR AR headset* OR holoLens OR VR headset*) \| 23,592 \| \| # 7 \| TS=(augment* AND virtual* AND mix* AND extend*) \| 128 \| \| # 6 \| TS=(AR NEAR VR NEAR MR NEAR XR) \| 15 \| \| # 5 \| TS=(extended reality OR extended-reality OR extend* realit*) \| 7,001 \| \| # 4 \| TS=(mixed reality OR mixed-realit* OR mix* realit* OR merged reality OR merged-realit* OR merged realit*) \| 10,066 \| \| # 3 \| TS=(augmented virtuality OR augment* NEAR virtual* OR augment* virtual*) \| 13,716 \| \| # 2 \| TS=(virtual reality OR virtual-reality OR immersive realit* OR virtual* realit* OR virtual reality systems) \| 57,655 \| \| # 1 \| TS=(augmented reality OR augmented-reality OR augmented reality system OR augment* realit* OR augment* NEAR realit*) \| 22,924 \| |
| --- | --- | --- | --- | --- | --- | --- | --- | --- | --- | --- | --- | --- | --- | --- | --- | --- | --- | --- | --- | --- | --- | --- | --- | --- | --- | --- | --- | --- | --- | --- | --- | --- | --- | --- | --- | --- | --- | --- | --- | --- | --- | --- | --- | --- | --- | --- | --- | --- | --- | --- | --- | --- | --- | --- | --- | --- | --- | --- | --- | --- | --- | --- | --- | --- | --- | --- | --- | --- | --- | --- | --- | --- | --- | --- | --- | --- | --- | --- | --- | --- | --- | --- | --- | --- | --- | --- | --- | --- | --- | --- | --- | --- | --- | --- | --- | --- | --- | --- | --- | --- | --- | --- | --- | --- | --- | --- | --- | --- | --- | --- | --- | --- | --- | --- | --- | --- | --- | --- | --- | --- | --- | --- | --- | --- | --- | --- | --- | --- | --- | --- | --- | --- | --- | --- | --- | --- | --- | --- | --- | --- | --- | --- | --- | --- | --- | --- | --- | --- | --- | --- | --- | --- | --- | --- | --- | --- | --- | --- | --- | --- | --- | --- | --- | --- | --- | --- | --- | --- | --- | --- | --- | --- | --- | --- | --- | --- | --- | --- | --- | --- | --- | --- | --- | --- | --- | --- | --- | --- | --- | --- | --- | --- |
