# Supplementary material for: Mixed Reality Platforms in Telehealth Delivery: Scoping Review
Source: JMIR Biomed Eng. 2023 Mar 24;8:e42709. doi: 10.2196/42709 (PMC11041465; doi:10.2196/42709)
Supplement: Multimedia Appendix 2 [file biomedeng_v8i1e42709_app2.docx]

**Multimedia Appendix 2**

**Table 1. Standard commercial AR/VR/MR hardware devices.**

|  | **Immersive Display Technologies** | **Reality Platform Type** |  |
| --- | --- | --- | --- |
|  | Google Glass (Google) | AR |  |
|  | Recon Jet (Recon Instruments) | AR |  |
|  | Laster WAVƎ headset (Laster Technologies) | AR |  |
|  | R-7 Smartglasses (Osterhout Design Group (ODG)) | AR |  |
|  | M300 Smartglasses (Vuzix) | AR |  |
|  | Haori Mirror (Haori Technology) | AR |  |
|  | BT-300, BT-350 (Epson) | AR |  |
|  | HoloLens, HoloLens2 (Microsoft) | AR & MR |  |
|  | Magic Leap One (Magic Leap) | AR & MR |  |
|  | Sony HMZ-T1, Sony HMZ-T2 (Sony) | VR |  |
|  | Occulus Rift, Oculus Go, Oculus Quest(Meta) | VR |  |
|  | GearVR (Samsung) | VR |  |
|  | HTC Vive (HTC) | VR |  |
|  | pico G2 4k (Pico) | VR |  |
|  | eSight 2, eSight 3 (eSight Corporation) | VR |  |
|  |  |  |  |
|  | **Non - immersive Display Technology** | |  |
|  | 3D Projectors (Generic) |  |  |
|  | Smartphones and Tablet Devices ( Android and IOS Support) |  |  |
|  | Standard computer, or laptop (High-end Specification) |  |  |
|  | 2D or 3D Television |  |  |
|  | Aquos Board PN-L603B (Sharp Electronics) |  |  |
