# Supplementary material for: Mixed Reality Platforms in Telehealth Delivery: Scoping Review
Source: JMIR Biomed Eng. 2023 Mar 24;8:e42709. doi: 10.2196/42709 (PMC11041465; doi:10.2196/42709)
Supplement: Multimedia Appendix 3 [file biomedeng_v8i1e42709_app3.docx]

**Multimedia Appendix 3**

**Table 1. Other relative hardware devices and systems used as a complete system.**

| Other hardware devices used in combination | |  |
| --- | --- | --- |
| **Optical capturing and tracking devices** | Kinect v2 , Kinect for Xbox One (Microsoft); Depth Camera D435 (Intel RealSense); PTZ Pro (Logitech); Ovrvision (Wizapply Team); THETA V (RICOH); VECTRA H1 (Canfield Scientific); EinScan-S ( SHINING 3D) |  |
|  |  |  |
|  |  |  |
| **Input devices** | Vive Controller or tracker (HTC); Oculus Controllers (Meta); Phantom Omni (Sensable Technologies); OS3D (Inertial Labs); Leap Motion Controller (ultraleap); Gloveone (NeuroDigital Technologies) |  |
|  |  |  |
|  |  |  |
| **Gaming Systems** | UE Ranger + BrightBrainer (Bright Cloud International Corp); Wii console and controllers (Nintendo) |  |
|  |  |  |
| **Biometric devices** | WristOx2® Model 3150 (Nonin); Digital pedometer ( Make - Not mentioned); Myo Armband (Thalmic Labs); Alpha wristband (Mio); Tymo plate (Tyromotion); MVN System (Xsens) |  |
|  |  |  |
|  |  |  |
| **Simulation models** | Phaco Eyes (Phillips Studio); SimMan 3G (Laerdal); Manikin (Make - Not mentioned); Plastic arm model (Make - Not mentioned); FLS box (Make - Not mentioned); 3-layer models consisting of skin, subcutaneous tissue, and bone (Make - Not mentioned) |  |
|  |  |  |
|  |  |  |
|  |  |  |
