# Supplementary material for: Mixed Reality Platforms in Telehealth Delivery: Scoping Review
Source: JMIR Biomed Eng. 2023 Mar 24;8:e42709. doi: 10.2196/42709 (PMC11041465; doi:10.2196/42709)
Supplement: Multimedia Appendix 4 [file biomedeng_v8i1e42709_app4.docx]

**Multimedia Appendix 4**

**Table 1. Software sources to constitute the telehealth and digital reality system.**

| **3D Modelling and Viewing application** | | TriDef 3D (TriDef); Blender version 2.67 (Blender Foundation); unity3d (Unity Technologies); Microsoft HoloLens 3D Viewer Beta (Microsoft Corporation); Panda3D (Carnegie Mellon University); Gazebo (Open Source Robotics Foundation); Cry Engine (Crytek ); Unreal Engine (Epic Games, Inc.); True 3D (EchoPixel, Inc.); Geomagic Design X (3D Systems, Inc.); Mixamo (Adobe Systems Incorporated.); cvi42 (Circle Cardiovascular Imaging Inc.); Holosurgery app |  |
| --- | --- | --- | --- |
|  |  |  |  |
|  |  |  |  |
|  |  |  |  |
|  |  |  |  |
|  |  |  |  |
|  |  |  |  |
|  |  |  |  |
| **Communication and Streaming Platforms** | | VSee (VSee); PubNub (PubNub); TeamViewer 6 (TeamViewer); HelpLightning (Help Lightning, Inc.); Pristine Eyesight® (PES) (Pristine, Inc.); Skype (Skype Technologies); Proximie (Proximie Limited); vTIME (vTime Limited); Xpert Eye platform (AMA XpertEye); Vuforia Chalk (PTC Inc.); Reacts application (INNOVATIVE IMAGING TECHNOLOGIES INC. and Reacts®); Onsite Connect (Librestream Technologies); Dynamics 365 Remote Assist (Microsoft Corporation); Thrive (Aetho); TeamViewer pilot (TeamViewer); WhatsApp (WhatsApp LLC); Photon Cloud (photon) |  |
|  |  |  |  |
|  |  |  |  |
|  |  |  |  |
|  |  |  |  |
|  |  |  |  |
|  |  |  |  |
|  |  |  |  |
|  |  |  |  |
| **File Transfer - Data center application services** | | Dropbox (Dropbox); Augmedix (Augmedix) |  |
|  |  |  |  |
| **Developed Software** | | H-TIME software (Tian et al); Stereoscopic AR Predictive Display or SARPD (Richter et al); Caps-Sim (Khalifa University); AUDIME system (CYBERNETICS LAB (EN)); STAR System (Rojas-Muñoz et al); TeleLNA (Related to Audime)VRRS (Follmann et al); ReHabgames(Ferreira and Menzes); (PR in VR) program (Jung et al); MoveHero Software (da Silva et al) |  |
|  |  |  |  |
|  |  |  |  |
|  |  |  |  |
|  |  |  |  |
| **Other specific application** | **Rehabilitation Games** | Jintronix system (Jintronix, Inc.); Optimov (Optimov); EaseVRx (AppliedVR, Inc.); Virtual Exercise Rehabilitation Assistant (Reflexion Health, Inc.) |  |
|  |  |  |  |
|  |  |  |  |
|  |  |  |  |
|  | **Health Management** | growlink™ patient app (Care Innovations PRA) |  |
|  |  |  |  |
|  | **Simulation Generator** | Matlab, Simulink (The MathWorks, Inc.) |  |
|  |  |  |  |
|  | **Screen Mirroring** | iDisplay (SHAPE GmbH) |  |
|  |  |  |  |
|  | **Audio editing** | QLab (Figure 53, LLC) |  |
|  |  |  |  |
